# Spatial and Temporal Variations in Pigment and Species Compositions of Snow Algae on Mt. Tateyama in Toyama Prefecture, Japan

**DOI:** 10.3389/fpls.2021.689119

**Published:** 2021-07-05

**Authors:** Tomomi Nakashima, Jun Uetake, Takahiro Segawa, Lenka Procházková, Akane Tsushima, Nozomu Takeuchi

**Affiliations:** ^1^Graduate School of Science, Chiba University, Chiba, Japan; ^2^Field Science Center for Northern Biosphere, Hokkaido University, Sapporo, Japan; ^3^Center for Life Science Research, University of Yamanashi, Kofu, Japan; ^4^Department of Ecology, Faculty of Science, Charles University, Prague, Czechia

**Keywords:** snow algae, red snow, pigment composition, astaxanthin, 18S rRNA, phylogenetic analysis, *Sanguina*, *Chloromonas*

## Abstract

Snow algae are photosynthetic microbes that inhabit the melting snow surface in alpine and polar regions. We analyzed the pigment and species composition of colored snow collected on Mt. Tateyama in Japan during the melting seasons of 2015 and 2016. High-performance liquid chromatographic analyses of the pigments extracted from the colored snow showed that their composition varied within the study area and were classified into four types: Type A (astaxanthin-monoester dominant), Type B (medium astaxanthin-monoester content), Type C (abundant primary carotenoids and free-astaxanthin), and Type D (abundant primary carotenoids and astaxanthin diesters). Types A and B were most commonly observed in the study area, whereas Types C and D appeared only at specific sites. Analysis of the 18S ribosomal RNA (18S rRNA) gene revealed six major amplicon sequence variants (ASVs) of snow algae, belonging to the *Sanguina, Chloromonas*, and *Chlainomonas* groups. The relative abundance of the algal ASVs showed that *Sanguina* was dominant (>48%) in both Types A and B, suggesting that the difference in astaxanthin abundance between the two types was caused by the production of pigments in the algal cells. The algal community structures of Types C and D differed from those of Types A and B, indicating that the primary carotenoids and astaxanthin diesters were derived from certain algal species in these types. Therefore, astaxanthin-rich *Sanguina* algae mostly induced the red snow that appeared widely in this alpine area; however, they were partially dominated by *Chloromonas* or *Chlainomonas* algae, causing different pigment compositions.

## Introduction

Snow algae are photosynthetic microbes that inhabit the melting snow surface in alpine and polar regions. Their blooms on the snow surface cause visible red- or green-colored snow because of various pigments in the algal cells. This phenomenon is observed worldwide, including in Japan, and is referred to as red or green snow. The blooms of snow algae can affect carbon and nitrogen cycles within the snowpacks and can also affect the melting rate of snow because of their light-absorbing effect (Onuma et al., 2020). Thus, it is important to understand the spatial distribution and the factors controlling the occurrence of algal blooms.

The pigments in snow algal cells are mainly chlorophylls and carotenoids, which have specific physiological functions in their cells (Bidigare et al., 1993; Müller et al., 1998). Chlorophylls play a role in photosynthesis and are present in active algal cells. However, carotenoids play a role in protecting cells from ultraviolet (UV) damage and potential photoinhibition, and transferring excitation energy to chlorophyll *a* (Takaichi, 2011). Carotenoids in algal cells are usually classified as primary and secondary carotenoids. Primary carotenoids, such as xanthophyll cycle pigments, are present in trace amounts in algal chloroplasts and are directly associated with photosynthesis. Secondary carotenoids, such as astaxanthin, are present in the algal cytoplasm and are more abundant than the primary carotenoids, and most exist as astaxanthin fatty acid esters (Remias and Lütz, 2007; Řezanka et al., 2008).

The abundance of pigments, in particular carotenoids, determines the visible coloration of algal cells, such as green, red, or orange, and varies seasonally and spatially on snowpacks. For example, astaxanthin and xanthophyll cycle pigments increased in algal cells of *Chloromonas nivalis* later during the melting season (Remias et al., 2010). Lutz et al. (2014) observed that white snow turned into red or green snow in 2 or 3 days on a Greenlandic glacier. The pigment composition of snow algae spatially varied in different locations in Svalbard and in different topographies, snow conditions, slope angles, and altitudes (Müller et al., 1998). Furthermore, astaxanthin contains various derivatives, and its composition in algal cells of *C. nivalis* has been reported to vary geographically in European Alps (Řezanka et al., 2013).

Variations in pigment compositions of colored snow are associated with algal species composition in the snow or with the relative abundance of carotenoid pigments controlled by the environmental conditions. One of the major groups of snow algae is the genus *Sanguina* (previously a part of *Chlamydomonas*), usually rich in astaxanthin in mature cyst cells and causes red-colored snow (Remias et al., 2005; Remias and Lütz, 2007; Holzinger et al., 2016). Another major group of snow algae is the genus *Chloromonas*, usually rich in primary carotenoids, such as zeaxanthin, violaxanthin, and secondary carotenoids, which causes green-colored snow or slightly brown orange-colored or pink-colored snow (Nedbalová et al., 2008; Remias et al., 2010).

Snow algae in colored snow usually consist of several species, and their community structure varies spatially in mountainous regions (Brown et al., 2016; Yakimovich et al., 2020). For example, *Sanguina* was dominant in red snow above the tree line, whereas *Chloromonas* was dominant in green- and orange-colored snow at lower elevations (Engstrom et al., 2020). In Antarctica, green-colored snow consists of *Chloromonas, Sanguina*, and *Chlorella*, usually rich in chlorophylls *a* and *b*, β-carotene, and lutein, whereas red-colored snow consists of *Chloromonas* alga containing abundant astaxanthin esters (Davey et al., 2019). The algal community of red-colored snow in Svalbard and Sweden consisted of *Chloromonas* and *Sanguina*; however, that of green-colored snow differed in two locations: *Microglena* sp. and *Raphidonema semperviens* were dominant in Svalbard, whereas *Sanguina* and *Chloromonas* were dominant in Sweden (Lutz et al., 2017).

Pigment composition also varies during the lifecycle of snow algae. For example, the cyst stage of algae contains abundant astaxanthin, whereas, in later stage, the flagellated green cells contain abundant primary carotenoids such as lutein (Osterrothová et al., 2019). *Sanguina* snow algae have more primary carotenoids during the earlier season, whereas they have astaxanthin during the later season (Remias et al., 2005).

The community structure and the pigment composition of snow algae can change depending on the physical or chemical conditions of the snow surface (Remias et al., 2005, 2010). Snow algae under conditions of strong ultraviolet radiation (UVR) or nutrient limitations produce more carotenoids and induce red-colored snow, whereas snow algae in weak radiation or rich nutrients produce fewer carotenoids and induce green-colored snow (Thomas and Duval, 1995; Leya et al., 2009). In particular, under limited nitrogen conditions, snow algal cells contain more abundant carotenoids (Britton and Edgar, 1998). However, Fujii et al. (2010) reported that astaxanthin-rich algae were found in snow with high nitrogen content. In contrast, Müller et al. (1998) showed no direct correlation between the secondary carotenoids of snow algal cells and the nutrient content of meltwater. Thus, there is still uncertainty between the environmental conditions and the pigment composition of snow algae. It is important to clarify the relationship between algal species and the pigment composition to understand their ecology on the snow surface.

Colored snow is widely observed on snowpacks in Japan and has been taxonomically studied in recent decades (Fukushima, 1963; Segawa et al., 2005; Muramoto et al., 2008; Tanabe et al., 2011; Matsuzaki et al., 2015; Terashima et al., 2017). Mt. Tateyama, an alpine area at elevations between 2,000 and 3,000 m above sea level (a.s.l.), is one of the snowiest mountains in Japan and is a place where colored snow frequently occurs during spring and summer. Red snow appears every year on the snow surface above the tree line at ~2,100 m a.s.l.; however, its distribution is spatially and seasonally heterogeneous in this alpine area. The variations in algal pigments and the community structure in this area are still not well-understood.

In this study, we analyzed the pigment composition and the 18S rRNA gene of snow algae in the colored snow collected from various sites in Mt. Tateyama during the melting seasons of 2015 and 2016. This study describes the spatial and temporal variations of algal pigments and the community structure in snowfields and discusses the relationships between species, life cycles, and pigments.

## Materials and Methods

### Study Site and Sample Collection

Mt. Tateyama is located in the Toyama Prefecture in the western part of Japan. It is a northern part of the Hida mountain range, one of the major mountain ranges on the main island (Honshu Island) of Japan, extending over 150 km from north to south. The elevation of the highest peak of Mt. Tateyama is 3,015 m a.s.l. Because the strong monsoon westerly blows from the Sea of Japan to the mountains during winter, heavy snow accumulates more than 5 m in depth in the mountain every year. The snow usually starts to melt in April and continues until the end of August. On the snow surface, red or green snow of algal blooming is commonly observed above the tree line (~2,100 m a.s.l.) from May to July every year.

**Figure 1:**
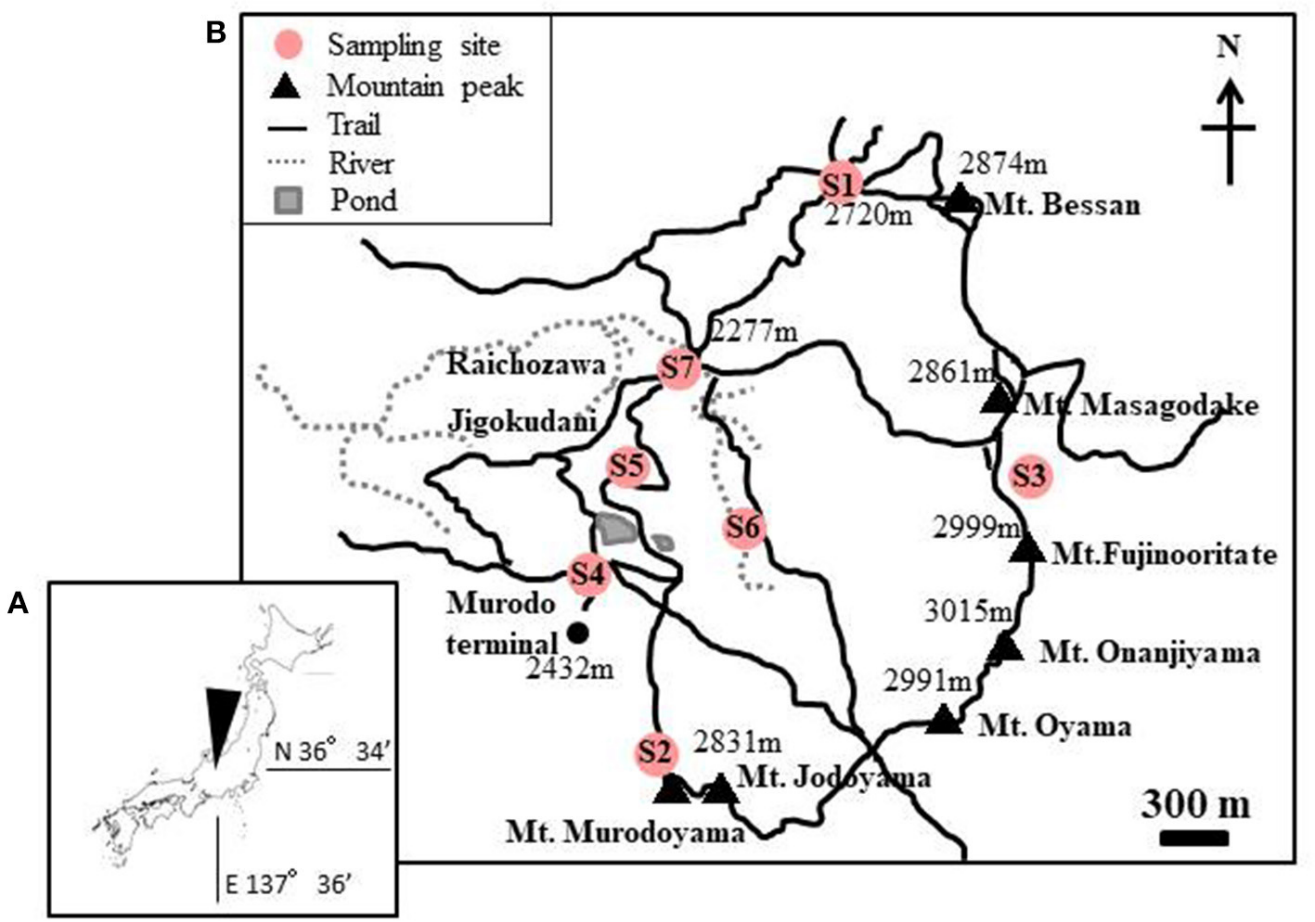
**(A)** Maps showing the location of the study area in Mt. Tateyama, Japan. **(B)** Locations of the sites where the red snow samples were collected. Numbers in the map indicate the altitude above sea level.

**Figure 2:**
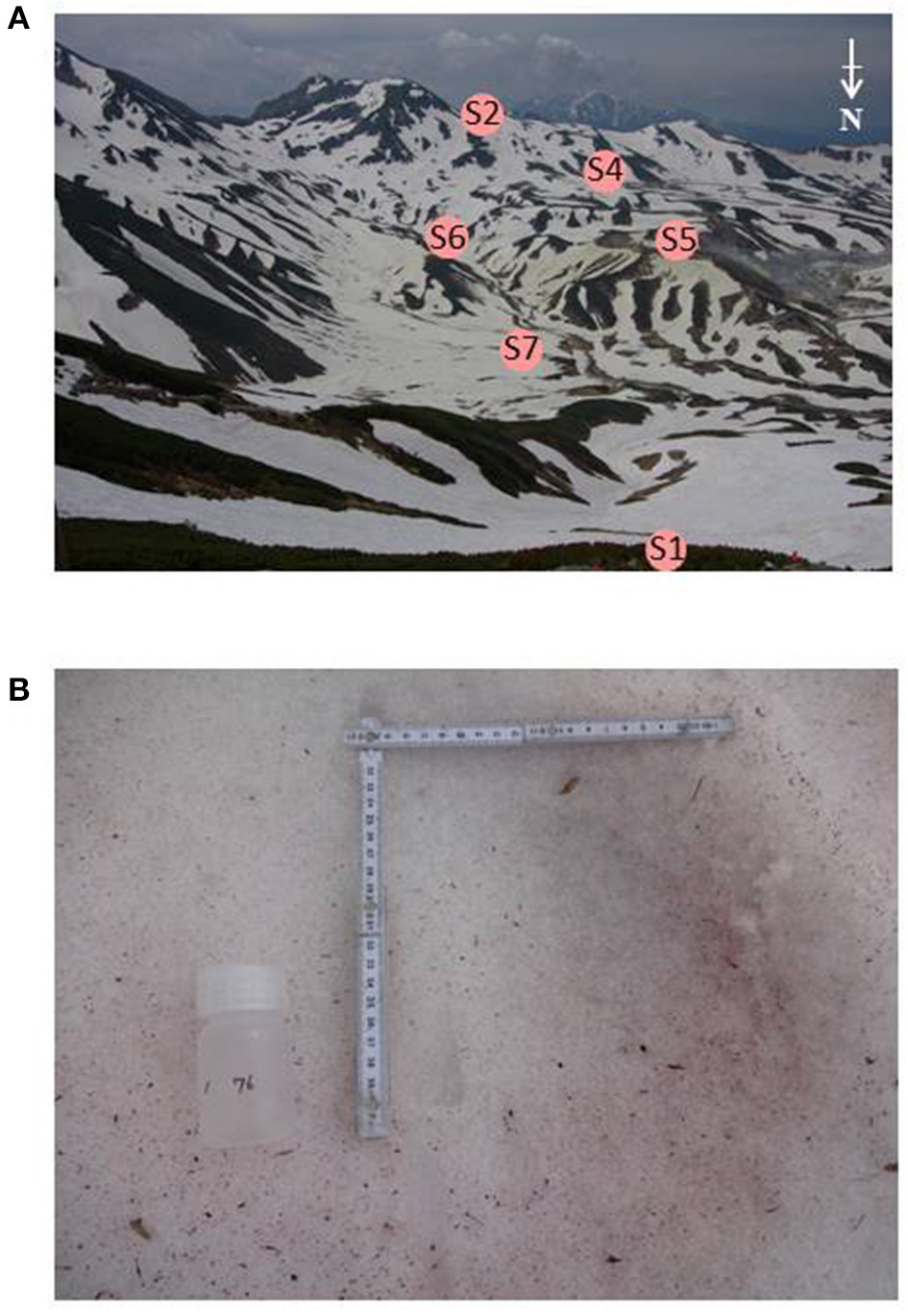
**(A)** Photographs of the study area, Murodo-Daira, alpine plateau, Mt. Tateyama. The site S3 is not visible from this shooting point. **(B)** Red snow surface at the site S6 observed in June 2015.

Snow samples were collected at seven sites in Murodo-Daira, a flat and old lava plateau in this alpine area at an elevation of ~2,400 m a.s.l (Figure 1, Table 1). Fieldwork was conducted in June and July of 2015 and June of 2016. We found visible red snow at all times and collected a total of 54 colored surface snow samples (Figure 2, Table 2). Red snow appeared widely in June 2015; however, it was patchy on a scale of ~10 cm in July 2015 and June 2016. The snow color varied slightly among the study sites; it was deep red and orange at S2 but red to brown at S7. The samples were transported in a frozen state to the laboratory at Chiba University and were stored in a freezer at −20°C until analysis. A part of the samples was kept at 0°C and used for microscopic observation. The algal cells in the samples were observed with an optical microscope (BX51, Olympus, Japan). Cell concentrations of each algal morpho-type were quantified by direct cell counting with the microscope.

**Table 1:** List of study sites showing the number of samples collected.

**Site No**.	**Elevation (m)**	**GPS coordinates**	**2015**		**2016**	**Total**
			**June**	**July**	**June**	
S1	2,720	N36.596614 E137.610712	1	1	0	2
S2	2,670	N36.568770 E137.600069	4	–	2	6
S3	2,650	N36.582900 E137.620111	3	–	–	3
S4	2,450	N36.578007 E137.597709	1	2	2	5
S5	2,360	N36.583555 E137.599125	1	3	0	4
S6	2,350	N36.581005 E137.605004	5	·	·	5
S7	2,300	N36.587690 E137.599254	10	12	7	29
Total			25	18	11	54

–*, No data; ·, No snow cover*.

**Table 2:** Number of samples classified into four pigment types at each study site and month.

**Site No. (m a.s.l.)**		**Pigment type**				**Total**			
		**A**	**B**	**C**	**D**	**A**	**B**	**C**	**D**
	June 2015	1	0	0	0				
S1	July 2015	1	0	0	0	2	0	0	0
(2,720)	June 2016	0	0	0	0				
	June 2015	4	0	0	0				
S2	July 2015	–	–	–	–	4	1	0	1
(2,670)	June 2016	0	1	0	1				
	June 2015	3	0	0	0				
S3	July 2015	–	–	–	–	3	0	0	0
(2,650)	June 2016	–	–	–	–				
	June 2015	0	1	0	0				
S4	July 2015	0	0	2	0	0	3	2	0
(2,450)	June 2016	0	2	0	0				
	June 2015	0	1	0	0				
S5	July 2015	0	3	0	0	0	4	0	0
(2,360)	June 2016	0	0	0	0				
	June 2015	2	3	0	0				
S6	July 2015	·	·	·	·	2	3	0	0
(2,350)	June 2016	·	·	·	·				
	June 2015	3	7	0	0				
S7	July 2015	4	8	0	0	12	17	0	0
(2,300)	June 2016	5	2	0	0				
Total						23	28	2	1

–*, No data; ·, No snow cover*.

### Analysis of Algal Pigments

Frozen snow samples (−20°C) were thawed in a refrigerator (4°C) before analysis. The meltwater of the samples was filtered through a glass fiber filter (Whatman Glass Microfiber Filters, GF/F, 25 mm). The filter was then placed in 6 mL of N, N′-dimethylformamide (DMF: density 99.5%) in an 8-mL polypropylene tube and left in the refrigerator for 24 h. The extracted pigments were stored at −20°C before analysis.

The extracted pigments in the DMF were quantified by high-performance liquid chromatography (HPLC; SHIMADZU LC-20A Series, Japan), composed of liquid chromatography (LC-20AT, Shimadzu), refractive index detector (RID-20A, Shimadzu), UV and visible detector (SPD-20A, Shimadzu), diode array detector (SPD-M20A, spectra from 300 to 800 nm, Shimadzu), solvent degasser, autosampler unit set at 4°C, and a column thermostat set at 60°C. The column [Phenomenex LUNA-C8 (2), 3 μm, 150 × 4.6 mm] is used a reverse-phase column. Solvent A contained 28 mM tetrabutylammonium acetate (1.0 M solution in water, Sigma-Aldrich)/methanol (HPLC grade, Wako) = 30/70 (v/v). Solvent B contained methanol (HPLC grade, Wako). The HPLC pump flow rate was 1 mL/min. The UV and visible detectors were set at 450 nm, and the diode array detector was set at 480 and 663 nm. Liquid chromatography and solvent were purged for 10 min before a new sample was injected. The injection contained 600 μL of solvent A and 400 μL of the filtered sample.

Analysis was undertaken with two methods in the different time sequences of the mixing ratio of the solvents.

Method 1 (percentage of solvent B): 0 min (20%)−18 min (45%)−65 min (90%)−66 min (95%)−71 min (95%)−72 min (20%)−82 min (20%).
Method 2: 0 min (20%)−10 min (50%)−15 min (80%)−45 min (95%)−48 min (20%)−53 min (20%).

Methods 1 and 2 were used for the samples collected in 2015 and in 2016, respectively.

Peaks that appeared in the chromatograms of 450 nm wavelength were identified and quantified with carotenoid and chlorophyll standards: chlorophyll *a*, chlorophyll *b*, lutein, β-carotene (3S, 3S′) astaxanthin, violaxanthin, antheraxanthin, zeaxanthin, canthaxanthin, echinenone, and pheophytin *a* (DHI Pigment Standards, Denmark). Chlorophyll *a* was quantified using the chromatograms of 663 nm wavelength since its peak often overlapped with those of antheraxanthin in the 450 nm chromatogram. Astaxanthin esters were quantified only for the trans-isomer in the 480 nm chromatogram using (3S, 3S′) astaxanthin standard. The standard was measured within 1 month before or after the sample was measured.

The pigment content of each sample quantified in this study is summarized in Supplementary Table 1. The hierarchical clustering of samples, as by calculating the Bray–Curtis dissimilarity index of pigment compositions, was conducted with R (version 4.0.5) and a VEGAN library (R Core Team, 2017).

### DNA Extraction

For samples collected in 2015, 10 mL of melted snow was filtered through a glass fiber filter (Whatman Glass Microfiber Filters, GF/F, 25 mm), combusted at 300°C for 1 h to remove DNA contamination before use. For samples collected in 2016, melted snow was directly added to the DNA extraction tube without filtration. DNA on the filter was extracted using a DNA extraction kit (FastDNA SPIN Kit for Soil, MP Biomedicals) by following the standard protocol of the manufacturer. Glass fiber filters were placed in lysing tubes directly and homogenized together with filtrates. The extracted DNA samples were stored at −30°C until subsequent analysis.

### 18S rRNA Gene Analysis by Illumina Sequencing

Partial 18S rRNA gene sequences (V4 region, ~380 bp) were amplified using primers Euk454F (5′-CCAGCASCYGCGGTAATTCC-3′) and EukR (5′-ACTTTCGTTCTTGATYRA-3′) (Logares et al., 2012). Polymerase chain reaction (PCR) mixture (20 μL) contained 1× KAPA *Taq* EXtra HotStart ReadyMix (Roche, Basel, Switzerland), 0.2 μM of each primer, and 1–5 μL template DNA. PCR was performed under the following cycling conditions: initial annealing at 95°C for 3 min, followed by 25–35 cycles of 95°C for 30 s, 50°C for 30 s, and 72°C for 60 s, and with a final extension at 72°C for 5 min. All PCR products were purified with AMPure XP (Beckman Coulter). As some of the PCR products included multiband or smeared bands, after agarose gel electrophoresis, PCR products were excised from the gel and purified using a NucleoSpin Gel and PCR clean-up kit (Macherey Nagel, Germany). The PCR products were labeled with a sample-unique index and Illumina adapter sequences at their 5′ end using the Nextera XT index kit v2 (Illumina, San Diego, CA, the United States). The PCR mixture (10 μL) contained 1× KAPA HiFi HS ReadyMix, 2 μL each of forward and reverse primers, and 1 μL of the recovered PCR products. PCR was performed under the following cycling conditions: 95°C for 3 min, followed by 8 cycles of 95°C for 30 s, 50°C for 30 s, and 72°C for 60 s, and with a final extension at 72°C for 5 min. All index PCR products were purified with AMPure XP and measured using a Qubit 2.0 Fluorometer (ThermoFisher Scientific) with Qubit dsDNA HS Assay Kit (ThermoFisher Scientific). Tagged amplicons were mixed with PhiX control DNA at a ratio of 80:20 and used as a template for MiSeq paired-end sequencing (2 × 300 bp) using Reagent kit v3 (Illumina) at the National Institute of Polar Research.

### Phylogenetic Analyses

All sequence libraries were clustered into amplicon sequence variants (ASVs) using the R package “DADA2” (Callahan et al., 2016). Taxonomy was assigned by the “assignTaxonomy” function (Wang et al., 2007) in DADA2 using the custom Silva 132 database (Quast et al., 2013). DADA2 removed all potential chimeric sequences. After removing identifiable plant sequences (order: Rosales, Sapindales, Pinales, Fagales, Lamiales), only Chlorophyta ASVs were filtered out, and the ASV composition was analyzed using the R package “Phyloseq” (McMurdie and Holmes, 2013). The taxonomy of ASVs was assigned using BLAST against the NCBI nr/nt database.

The 18S rDNA alignment of snow-inhabiting *Chloromonas/Chlainomonas* contained 47 sequences (1,567 bp) examined in the previous studies (e.g., Matsuzaki et al., 2019; Procházková et al., 2019a), as well as five ASVs (377 bp long) generated in this study (ASV2-ASV6), and the 18S rDNA matrix focused on *Sanguina* spp. consisted of 30 sequences (1,581 bp) published before (e.g., Procházková et al., 2019b), as well as one ASV (378 bp long) acquired in this study (ASV1). For the former dataset, the mesophilic species of the *Chloromonas* clade (Pröschold et al., 2001) or the *Chloromonadinia* clade (Nakada et al., 2008) were selected as the outgroup; and for the latter dataset, the mesophilic species of Reinhardtii clade sensu (Pröschold et al., 2001) were selected as the outgroup. The best-fit nucleotide substitution model was estimated using jModeltest 2.0.1 (Posada, 2008). Based on the Akaike information criterion, the TIM2+I+G and TIM3+I+G models were selected for the former and latter datasets, respectively. The phylogenetic tree of 18S rDNA was inferred by Bayesian inference (BI) using MrBayes (version 3.2.6) (Ronquist et al., 2012). Two parallel Markov chain Monte Carlo runs for 3,000,000 generations with one cold and three heated chains were conducted for both alignments using the selected best-fit evolutionary models, with trees sampled every 100 generations. The first 25% were discarded as burn-in. The maximum-likelihood (ML) phylogenetic tree was constructed using GARLI 2.0 (Zwickl, 2006). ML analysis consisted of rapid heuristic searches (100 pseudo-replicates) using automatic termination (genthreshfortopoterm command set to 100,000). The convergence of the two cold chains was checked by the average standard deviation of split frequencies (0.000868 and 0.000384 for the former and latter datasets, respectively). Bootstrap analyses and Bayesian posterior probabilities were used to assess the support of the clades. Values of posterior probabilities and bootstrap support were treated as weak (BI < 0.5, ML < 50%), moderate (BI 0.54-0.94, ML 50-79%) and high (BI > 0.94, ML > 79%) (Skaloud and Peksa, 2010).

### Data Availability

Raw sequence data are available from BioProject: PRJNA726817 in the Sequence Read Archive of National Center for Biotechnology Information (NCBI; https://www.ncbi.nlm.nih.gov/sra/). Sequences of 6 major ASVs are deposited in NCBI Genbank (Accession number: MZ317533 - MZ317538).

## Results

### Microscopic Observation of Algal Cells

**Figure 3:**
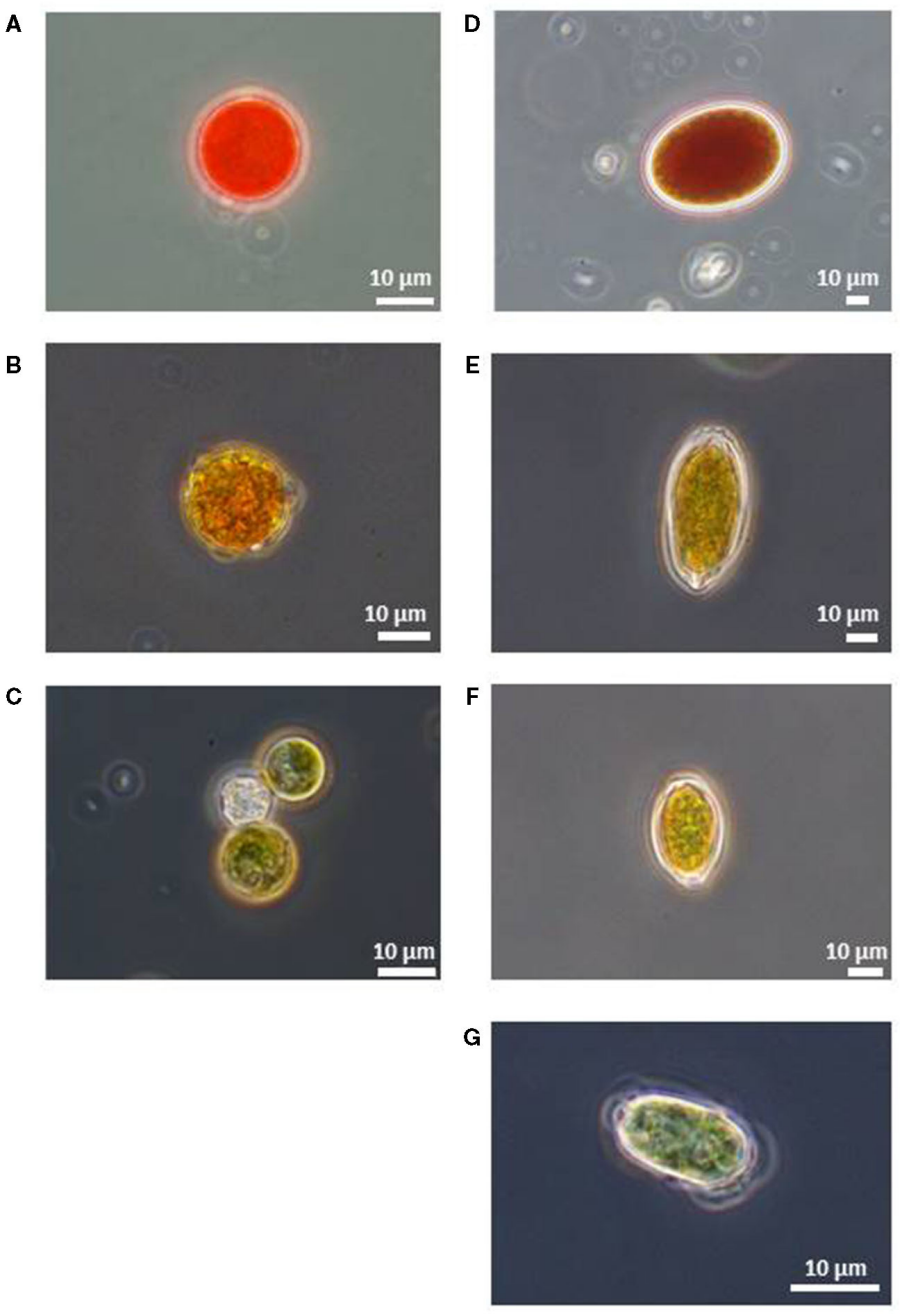
Microscopic photographs of snow algal cells in red snow samples collected in Mt. Tateyama (reproduced from Nakashima and Takeuchi, 2017). **(A)** Red spherical cell, **(B)** Orange spherical cell, **(C)** Green spherical cell, **(D)** Red ellipsoidal cell, **(E)** Orange oval cell with cell wall flanges, **(F)** Yellow oval cell with cell wall flanges, and **(G)** Green oval cell.

Microscopic observation revealed that all of the red snow samples contained algal cells of various colors and cell shapes as described previously by Nakashima and Takeuchi (2017). The algal cells were often attached to small mineral particles. The algal cells were roughly classified into seven types based on the color and morphology: (1) Red or deep red spherical cells with thick cell walls (Figure 3A). Chloroplasts were observed at the center of the cell. The size of cells was 23.7 ± 6.0 μm in diameter. (2) Light red or orange spherical cells with thick cell walls (Figure 3B). The size of cells was 20.8 ± 3.2 μm in diameter. (3) Green and small-sized spherical cells (Figure 3C). The size of cells was 13.8 ± 2.6 μm in diameter. (4) Deep red ellipsoidal cells, with smooth cell wall (Figure 3D). The size of cells was 46.9 ±7.6 μm in the major axis and 36.7 ± 5.1 μm in the minor axis. (5) Orange-colored oval cells, with cell wall flanges (Figure 3E). The size of cells was 44.4 ± 5.9 μm in the major axis and 24.1 ± 2.6 μm in the minor axis. (6) Yellow-colored and spindle-shaped cells (Figure 3F). The cell size was 27.4 ± 5.0 μm in the major axis and 16.0 ± 2.1 μm in the minor axis, smaller than (5) and larger than (7). (7) Green-colored oval cells (Figure 3G). Whole cell or center of the cell was colored light green and oval. The size of cells was 16.7 ± 3.1 μm in the major axis and 10.6 ± 1.9 μm in the minor axis.

The relative abundance of these cell types varied among the samples. Red, orange, and green spherical cells and green oval cell were contained in almost all samples in this study area. The other cell morphology appeared only at the specific site. Red ellipsoidal cells were included only in the samples at the site S2. Orange oval cell included in the samples at the sites S2, S4, and S7. Yellow oval cell was included in the samples at the sites S2 and S4; however, its abundance was relatively small.

### Algal Pigment Composition

**Figure 4:**
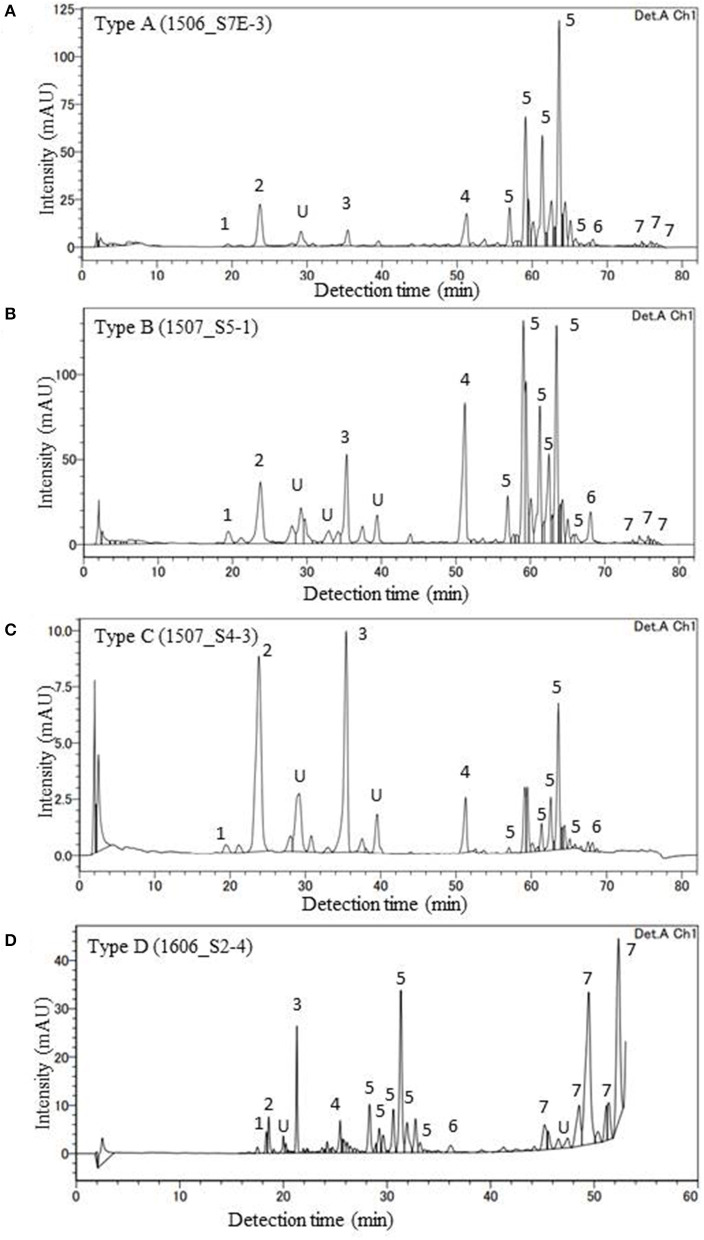
HPLC chromatograms of four major pigment types of red snow samples (**A**: Type A, **B**: Type B, **C**: Type C, **D**: Type D) in this study (detection wavelength: 450 nm). Peaks with numbers or alphabet in the chromatograms indicate the algal pigments identified in this study. 1: Violaxanthin, 2: Astaxanthin, 3: Lutein, 4: Chlorophyll *b*, 5: trans-Astaxanthin (monoester), 6: β-carotene, 7: trans-Astaxanthin (diesters), and U: Unknown.

HPLC analysis of algal pigments revealed that the algal snow collected in this contained mainly two chlorophylls (chlorophyll *a* and chlorophyll *b*) and primary and secondary carotenoids (violaxanthin, lutein, β-carotene, and astaxanthin) (Figure 4). Astaxanthin included mainly three different forms: 3S, 3S′ trans-astaxanthin (free-astaxanthin), astaxanthin-monoester, and astaxanthin diesters. The astaxanthin esters showed several peaks in the chromatogram.

**Figure 5:**
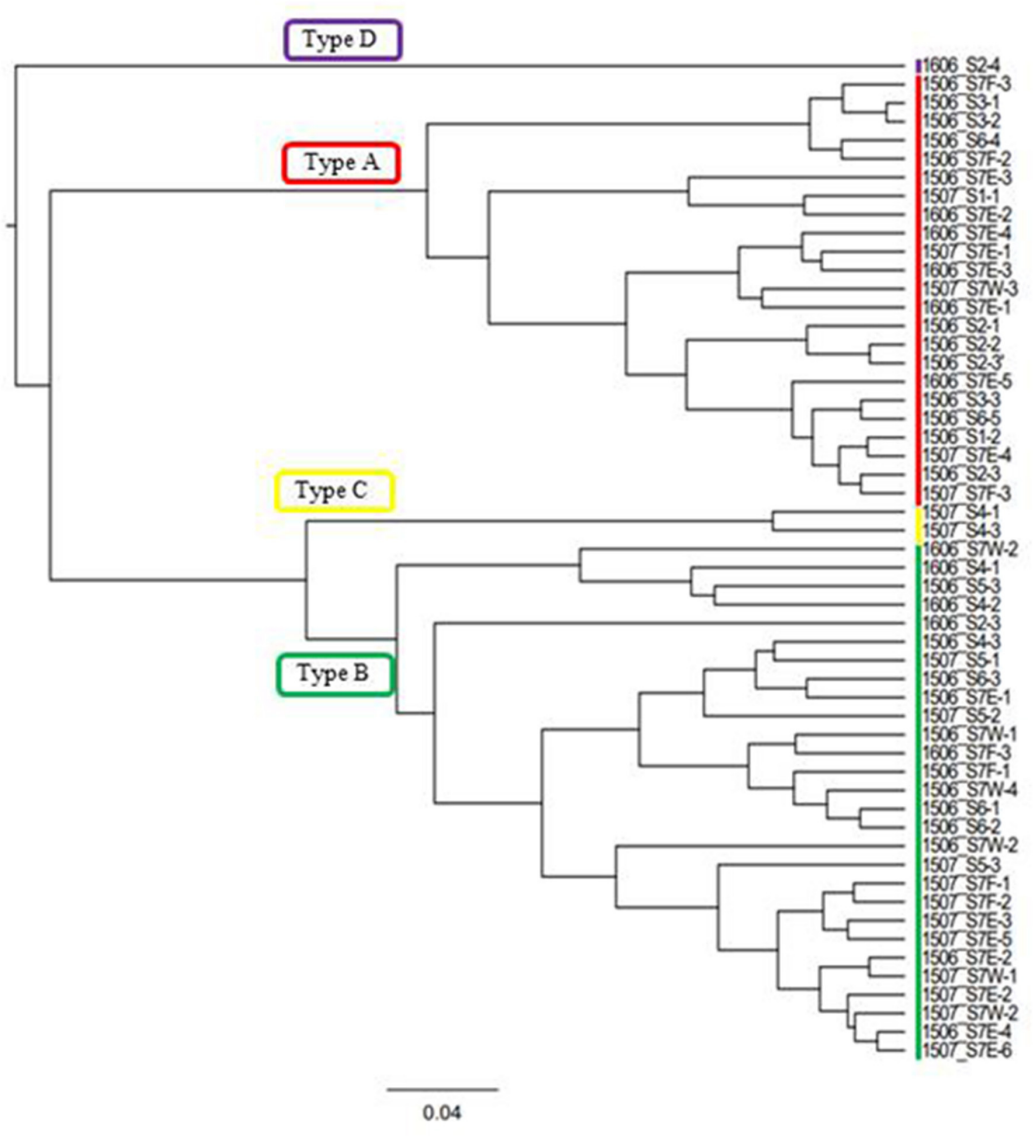
Cluster analysis performed based on the algal pigment composition of red snow samples. Four groups classified in this study based on the result were shown (Types A, B, C, and D).

**Figure 6:**
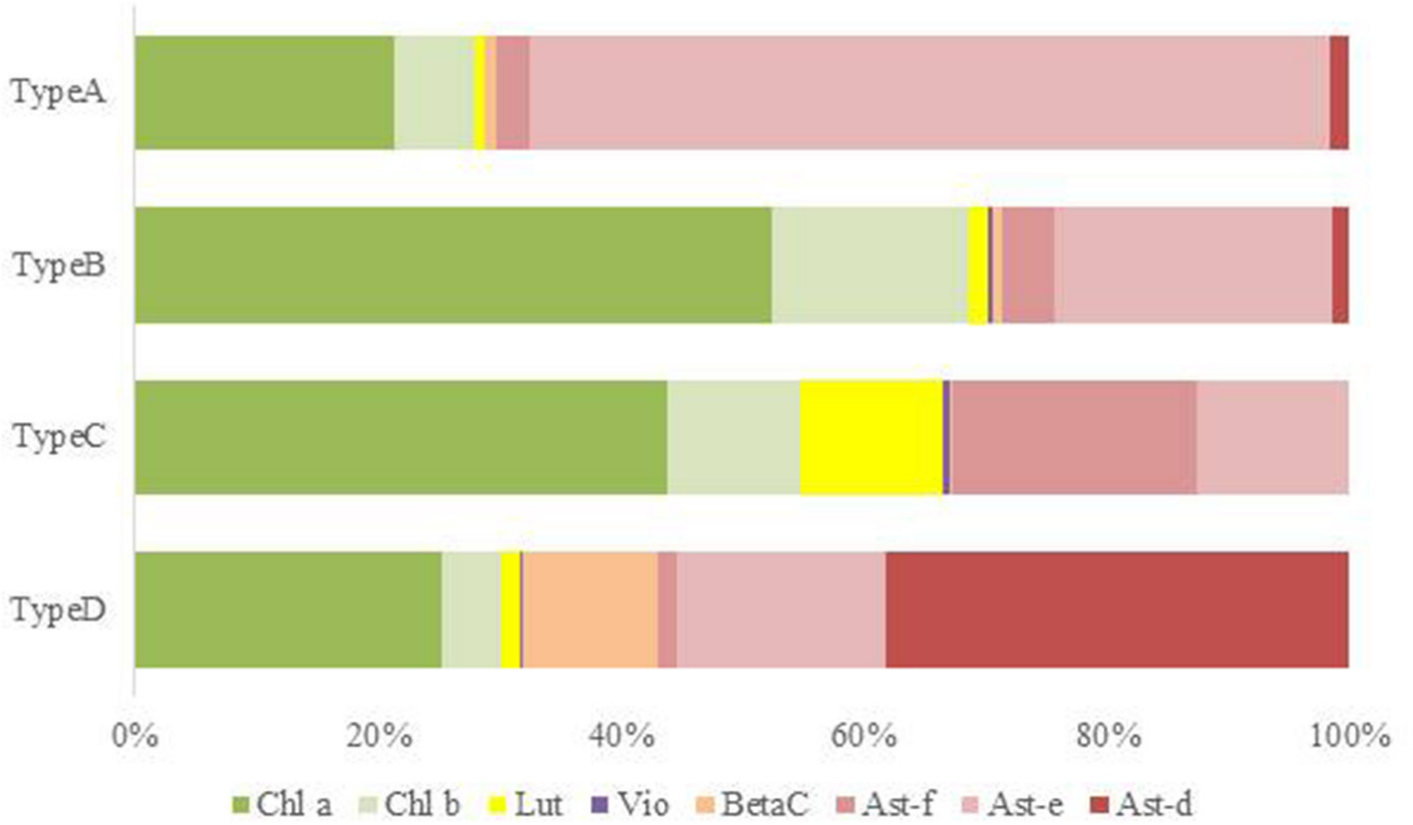
Mean pigment composition of four types of red snow samples in Mt. Tateyama. Abbreviations: Chl a: chlorophyll a; Chl b: chlorophyll b; Lut: lutein; Vio: violaxanthin; BetaC: β-carotene; Ast-f: free-astaxanthin; Ast-e: astaxanthin-monoester; Ast-d: astaxanthin-diesters.

In this study, the composition of these pigments varied among the samples. Based on the cluster analysis, the samples can be classified into four pigment types (Types A, B, C, and D as shown in Figure 5). Figure 6 shows the mean proportion of each pigment for the four pigment types. Type A was characterized by the dominance of astaxanthin-monoester, which accounted for 43.4–76.3% (mean: 63.6%) of the total pigments. Type B was characterized by the medium astaxanthin-monoester content, which accounted for 7.5–35.1% (mean: 22.0%) of the total pigments. The primary carotenoids were in trace levels in both Types A and B. Type C was characterized by the relatively high abundance of free-astaxanthin and primary carotenoids, mainly lutein. The mean abundance of free-astaxanthin and the mean abundance of lutein were 20.0 and 11.6%, respectively, of the total pigment. Type D was characterized by the high abundance of astaxanthin diesters, which accounted for 38.1% of the total pigment. The number of samples classified as Types A, B, C, and D were 23, 28, 2, and 1, respectively (Table 2).

The mean compositions of algal cell morphology in the four pigment types are shown in Supplementary Figure 1. The red, orange, and green spherical cells were dominant in both Types A and B (>90%), however red spherical cells was more abundant in Type A than Type B. Type C was dominated by the orange oval cell, accounting for 84% in the total cells. Type D was dominated by the red ellipsoidal cell, accounting for 89% in the total cells.

### Spatial Variation in Pigment Types

Table 2 shows the number of samples of the four pigment types in each study site, indicating that the pigment types varied spatially in the study area. Types A and B were most commonly observed in this area: Type A occurred at five sites (S1, S2, S3, S6, and S7) and Type B occurred at five sites (S2, S4, S5, S6, and S7) among seven study sites. Types C and D occurred only at sites S4 and S2, respectively. There was almost no difference in the appearance of pigment type among the different months of sample collection. Types A and B were always observed at site S7 in all field investigations (June 2015, July 2015, and June 2016).

### Phylogenetic Analyses of the 18S rRNA Gene of the Snow Algae

**Figure 7:**
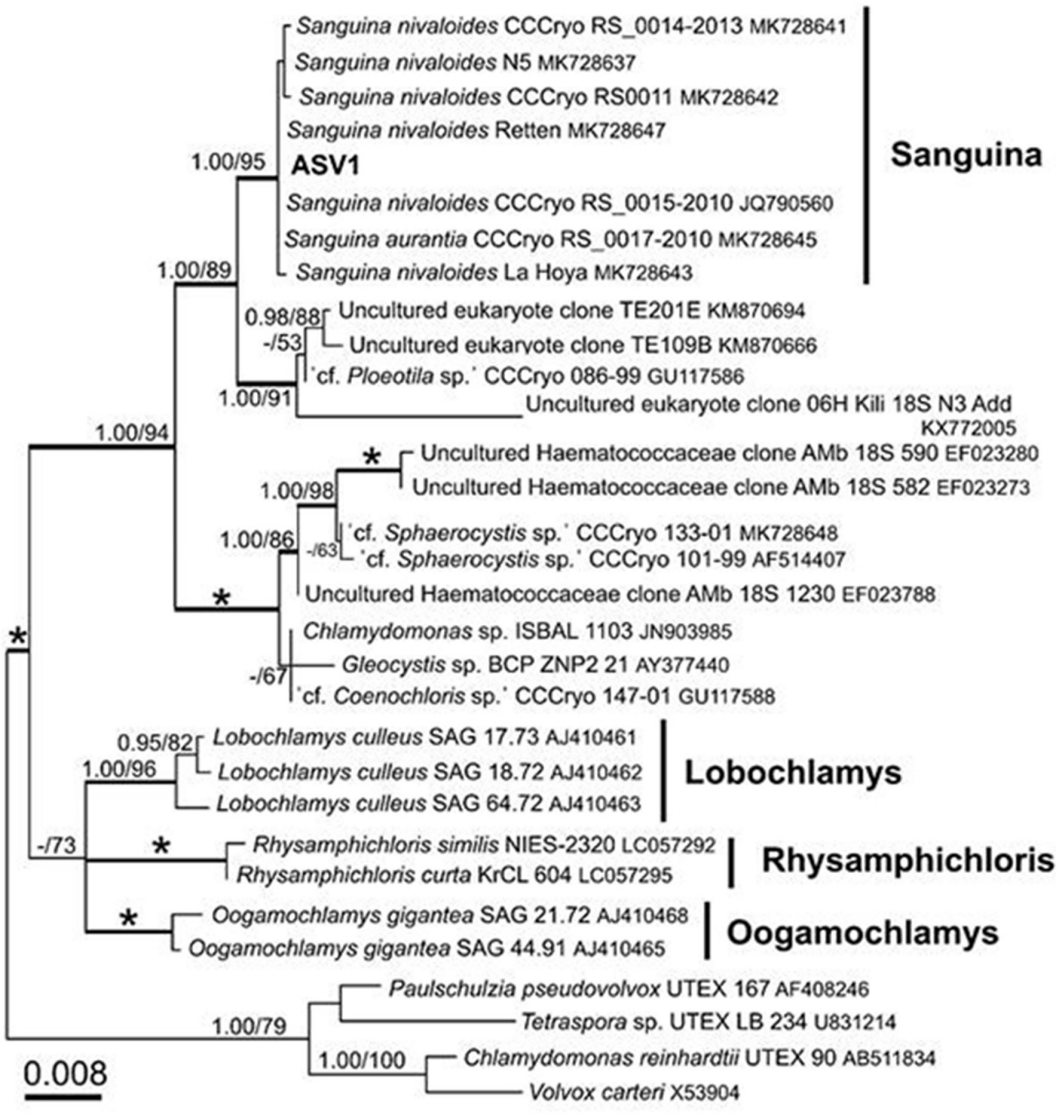
18S rRNA-gene-based maximum likelihood phylogeny of *Sanguina* spp. showing phylogenetic position of one of the major ASV reads (ASV1) detected in the red snow samples in Mt. Tateyama, Japan. Posterior probabilities (≥0.95) and bootstrap values from maximum likelihood analysis (≥50%) are shown. Full statistical support (1.00/100) is marked with an asterisk. Thick branches represent nodes receiving the highest posterior probability support (1.00). Accession numbers, strain, or field sample codes are indicated after each species name.

**Figure 8:**
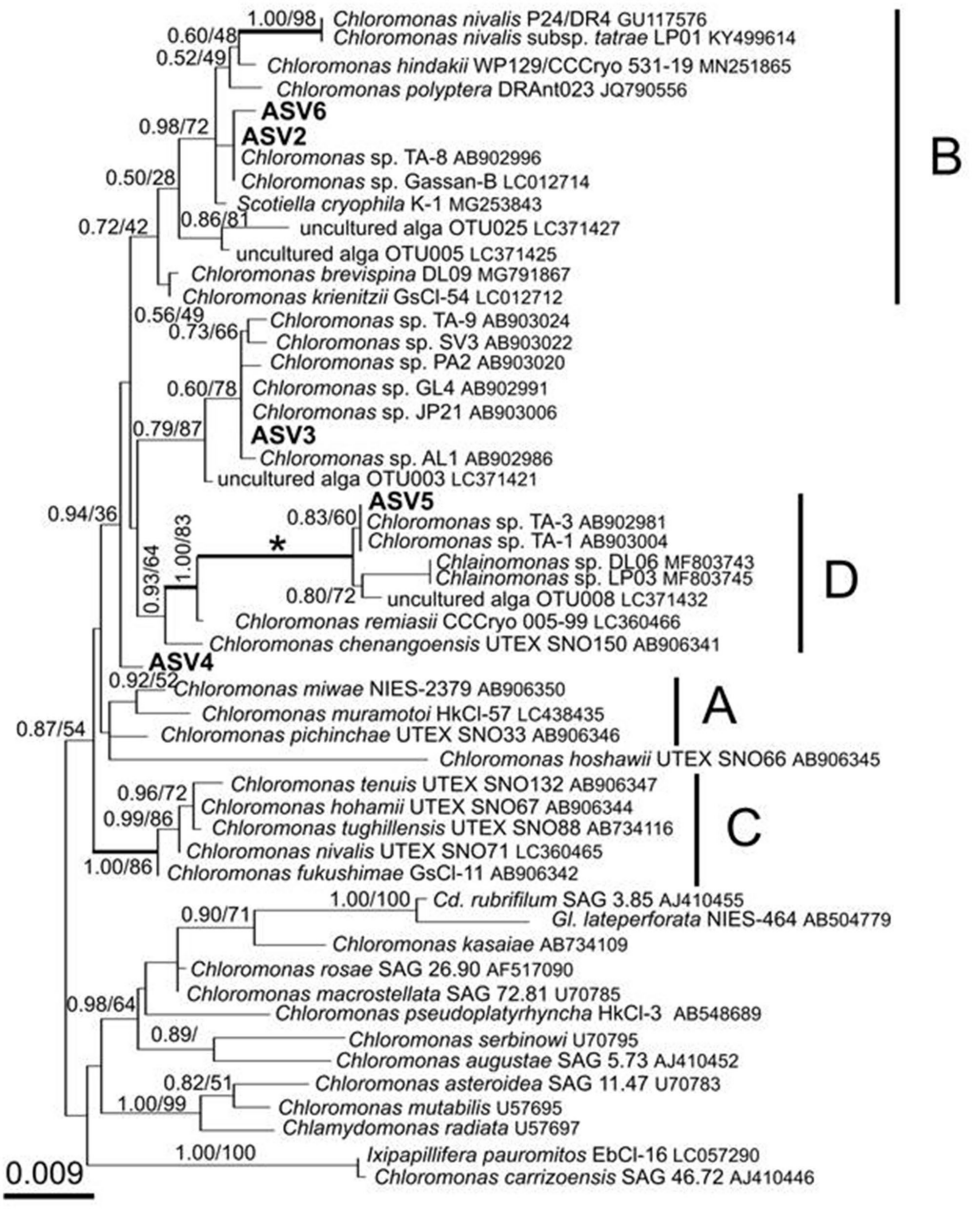
18S rRNA-gene-based maximum-likelihood phylogeny of snow-inhabiting *Chloromonas/Chlainomonas* spp. showing phylogenetic positions of the five major ASV reads (ASV2–ASV6) detected in the red snow samples in Mt. Tateyama, Japan. The labeled clades “A,” “B,” and “C” correspond to Matsuzaki et al. (2019). Posterior probabilities and bootstrap values from maximum likelihood analysis are shown. Full statistical support (1.00/100) is marked with an asterisk. Thick branches represent nodes receiving the highest posterior probability support (1.00). Accession numbers, strain, or field sample codes are indicated after each species name.

After quality filtering, the 18S rRNA gene amplicon sequence reads from the red snow comprised 2,568,681 high-quality paired-end sequence reads, which were clustered into 689 ASVs, with an average amplicon length of 381.52 bp (Supplementary Table 2). Classification of the sequence data revealed that Chlorophyceae dominated the snow algal communities (99.9% in the total Chlorophyta). Chlamydomonadales contained major 6 ASVs, and the observed number of ASVs in Chlamydomonadales were 15, 18, and 24 ASVs for the samples from June 2015, July 2015, and June 2016, respectively (Supplementary Table 3). The most abundant ASV was ASV1 (MZ317533), accounting for 45.6% of Chlorophyta sequences, it was closely related to *Sanguina nivaloides* CCCryo RS 0015-2010 (JQ790560) and *Sanguina aurantia* CCCryo RS 0017-2010 (MK728645) classified in the highly/highly (BI/ML) supported *Sanguina* clade (Figure 7). The second abundant ASV was ASV2 (MZ317534), accounting for 32.1% of Chlorophyta sequences, and it was a member of highly/moderately supported subclade of the *Chloromonas* clade-B (Figure 8). ASV2 was closely related to snow algae and consists of *Chloromonas hindakii* WP129/CCCryo 531-19 (MN251865), *Chloromonas polyptera* DRAnt023 (JQ790556), *Chloromonas* sp. Gassan-B (LC012714), and *Chloromonas* sp. TA-8 (AB902996) (Table 3).

**Table 3:** Top six ASVs (377-378 bp long) of 18S rRNA genes aligned and assigned to Chlorophyta in the samples, the closest relatives of the 18S rRNA genes that are based on the Nucleotide BLAST search in NCBI, and the sequence identity of 18S rRNA gene (%).

**ASV No**.	**Relative abundance (%)**	**Closest identified relatives**	**Accession numbers**	**Sequence identity (%)**
ASV 1	45.6	*Sanguina nivaloides* CCCryo RS 0015-2010	JQ790560.1	100
		*Sanguina aurantia* CCCryo RS 0017-2010	MK728645.1	100
ASV 2	32.1	*Chloromonas hindakii* WP129/CCCryo 531-19	MN251865.1	100
		*Chloromonas polyptera* DRAnt023	JQ790556.1	100
		*Chloromonas* sp. Gassan-B	LC012714.1	100
		*Chloromonas* sp. TA-8	AB902996.1	100
ASV 3	7.6	Uncultured alga: OTU003	LC371421.1	100
		*Chloromonas* sp. TA-9	AB903024.1	100
		*Chloromonas* sp. SV-3	AB903022.1	100
		*Chloromonas*_sp_PA2	AB903020.1	100
		*Chloromonas*_sp_GL4	AB902991.1	100
		*Chloromonas*_sp_JP21	AB903006.1	100
		And many others		
ASV 4	3.6	Uncultured alga: Otu025	LC371427.1	99.2
		Uncultured alga: Otu005	LC371425.1	99.2
		Uncultured alga: Otu003	LC371421.1	99.2
		*Chloromonas* sp. TA-9	AB903024.1	99.2
		*Chloromonas* sp. SV-3	AB903022.1	99.2
		And many others		
ASV 5	3.5	*Chloromonas*_sp_TA_1	AB903004.1	100
		*Chloromonas*_sp_TA_3	AB902981.1	100
		Uncultured alga: OTU008	LC371432.1	99.5
		*Chlainomonas* sp. LP03	MF803745.1	98.7
		*Chlainomonas* sp. DL06	MF803743.1	98.7
ASV 6	2.5	*Chloromonas hindakii* WP129/CCCryo 531-19	MN251865.1	99.7
		*Chloromonas polyptera* DRAnt023	JQ790556.1	99.7
		*Chloromonas* sp. Gassan-B	LC012714.1	99.7
		*Chloromonas* sp. TA-8	AB902996.1	99.7

*Top six ASVs were chosen which contained more than 1% in total ASVs*.

The third most abundant ASV was ASV3 (MZ317535), representing 7.6% of Chlorophyta sequences reads and belonged to an unnamed moderately/highly supported *Chloromonas* clade. It consisted of uncultured alga OTU003 (LC371421.1), uncultured *Chloromonas* sp. TA-9 (AB903024), uncultured *Chloromonas* SV-3 (AB903022), etc. (Figure 8).

The ASV5 (MZ317537), representing 3.5% reads of Chlorophyta sequences, was a member of the *Chloromonas* clade-D (Figure 8) and closely related to *Chlainomonas* sp. DL06/LP03 (MF803743, MF803745). ASV5 was 100% identical with the two algae tentatively assigned as “*Chloromonas*_sp_TA_1” (AB903004) and “*Chloromonas*_sp_TA_3” (AB902981) by single cell sequencing.

**Figure 9:**
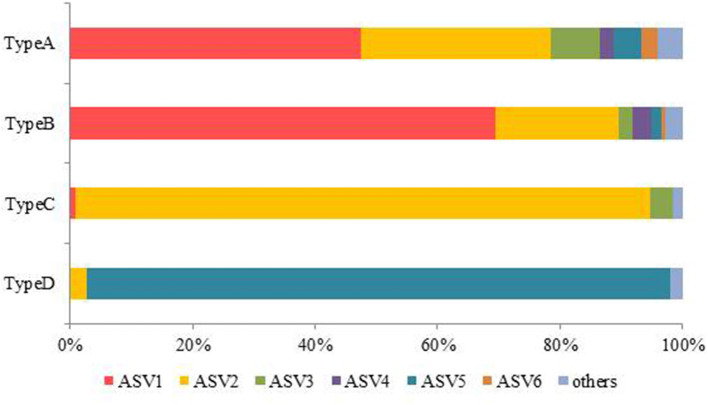
Mean relative abundance of algal ASVs containing the four pigment types.

Figure 9 shows the relative abundance of the ASVs of snow algae among the four pigment types. Both Types A and B were dominated by ASV1 and ASV2. ASV1 was most dominant in Types A and B, accounting for 48 and 69%, respectively. ASV2 was secondary dominant in the types, accounting for 31 and 20%, respectively. Types C and D were dominated by ASV2 (94%) and ASV5 (95%), respectively.

## Discussion

### Red Snow Commonly Appeared in This Area (Types A and B)

Types A and B, which were dominated by the secondary carotenoid of astaxanthin-monoester, were the most common types of red snow in this study area, suggesting that most of the red snow is due to snow algae containing abundant astaxanthin-monoester. Phylogenetic analysis showed that the dominant ASV in both Types A and B was ASV1 was a part of *Sanguina*-clade in the 18S rRNA gene phylogenetic tree (formerly assigned to *Chlamydomonas*, Procházková et al., 2019b). As *Sanguina* sp., a typical snow alga distributed worldwide, has been reported as an astaxanthin-rich snow alga (Remias et al., 2005), it is reasonable to suggest that the red snow of Types A and B was dominated by *Sanguina* sp. rich in astaxanthin.

Types A and B were both dominated by ASV1; therefore, the difference in the abundance of astaxanthin between these two types was probably due to the astaxanthin content in each algal cell. In Type A, algal cells exhibited higher astaxanthin-to-chlorophyll *a* ratio (range: 1.31–6.20, mean: 3.3:1), whereas algal cells in Type B had a lower astaxanthin-to-chlorophyll *a* ratio (range: 0.16–0.92, mean: 0.54:1). A previous study suggested that the pigment composition in the *Sanguina* group alga cells varied depending on the environmental conditions—for example, abundant production of astaxanthin under intense UV radiation (Remias et al., 2005) or under nitrogen-limited conditions (Lutz et al., 2016). Also the ratio of astaxanthin to chlorophyll *a* varied with the individual extent of maturation of a population in course of the summer season (Procházková et al., 2021). In this study, at the higher elevation sites, only Type A appeared (S1, S2, and S3), whereas in the lower elevation sites both Types A and B appeared (S6 and S7), and only Type B appeared at the low elevation site (S2, S4, and S5). At the higher elevation sites, the melt rate of snow was probably lower because the air temperature was lower than at lower elevations. The increase in astaxanthin in cells benefits snow surface-dwelling hypnoblasts (Dial et al., 2018) and is consistent with this conclusion. Furthermore, all the sites were located near the mountain ridge, where there was less vegetation due to windy conditions. The abundance of astaxanthin in the algal cells of ASV1 may be affected by the environmental conditions of the snow surface and cell maturation process.

Since the fragment of 18S rDNA (this study) or even the whole length of 18S rDNA is insufficient to distinguish the different *Sanguina* species (i.e., *S. nivaloides* and *S. aurantia*) (Procházková et al., 2019b), the distinct pigment compositions of Types A and B are also possibly due to abundance of different *Sanguina* species. Microscopy showed that, red and orange spherical cells contained in the samples of Types A and B were morphologically very similar to the mature cyst of *S. nivaloides* and *S. aurantia*, respectively, however, the size of the orange cells was significantly larger than that of *S. aurantia* (Procházková et al., 2019b, 2021). Thus, we cannot exclude that an undescribed *Sanguina* sp. may occur in snow in Mt. Tateyama.

### Red Snow of Special Properties Appeared in Specific Sites (Types C and D)

Pigment Type C, which is rich in primary carotenoids, including lutein and free-astaxanthin, appeared only at the site S4 in this study area, suggesting that special conditions induce this type of red snow at the site. However, we did not find any specific chemical (pH or EC) or topographical conditions at this site. When we collected the sample, the snow depth at this site was shallower (<20 cm) compared with those at the other sites (more than 50 cm). DNA analysis showed that this type was mostly dominated by ASV2, indicating that the primary carotenoids in Type C were derived from the algal cells of ASV2. ASV2 was one of the common algal species in this study area, as it was detected in many samples, including in Types A and B samples The phylogenetic tree showed that ASV2 was closely related to *Chloromonas* spp., which is consistent with their dominant pigments. Previous studies have shown that cells of *C. nivalis* have abundant primary carotenoids such as xanthophyll cycle pigments and lutein, which is distinctive from *Sanguina* sp. that contains mainly secondary carotenoids such as astaxanthin (Remias et al., 2010). Therefore, this type of snow probably has conditions to be dominated by the *Chloromonas* alga.

Pigment Type D, which was characterized by abundant astaxanthin diesters, was dominated by ASV5, indicating that the pigment was derived from this algal species. The lack of ASV1 (*Sanguina* sp.) in this snow type was distinctive from the other types in this study. This type appeared only at the site S2, suggesting special conditions induced this type of red snow at the site. Astaxanthin in algal cells is present in various molecular forms and often combines with one or two fatty acids and glucose. Fatty acids play an important role in cell survival at low temperatures (Řezanka et al., 2013). A previous study revealed that such molecular forms of astaxanthin in algal cells were changed by species. For example, the algal cells of the *Sanguina* group, which have abundant astaxanthin, mainly contained monoesters in the stage of resting spores, whereas the algal cells of *Chlainomonas* group were mainly present as astaxanthin diesters in the vegetative cell stage (Bidigare et al., 1993, Remias et al., 2016). The phylogenetic tree showed that ASV5 was closely related to *Chlainomonas* sp. and algal cell morphology in Type D was distinctive from other pigment types—red ellipsoidal cell dominant resembled in cell size, cell shape and pigment colouration of snow *Chlainomonas*. The snow surface at this site may be a suitable condition for the growth of this alga in this region. The site S2 was located on a southern mountain ridge in this study area; however, we did not find any other specific chemical (pH or EC) or topographical conditions at this site.

### Variations in the Algal Community in Red Snow in Tateyama Mountain Region

Our results showed that the community structure of red snow in the Tateyama Mountains area varied seasonally and spatially among the sites and largely affected the pigment composition. The presence of diverse algal communities within a relatively small mountainous area suggests that the algal community of red snow is determined by local environmental conditions and the migration process of algal cells to the snow surface.

Most of the red snow in this area is dominated by ASV1, *Sanguina* sp., a typical algal species in red snow worldwide. The previous study showed the presence of cosmopolitan species of snow algae, which appeared in the Arctic and Antarctic regions, and *Sanguina* [i.e., “*Chlamydomonas”*-snow group B) was one of them (Segawa et al., 2018)]. The snow alga *Sanguina* sp. observed in the red snow in this study may have originated from common sources of the world and migrated from the atmosphere onto the snow surface. However, another study described that the *Sanguina* snow alga in red snow does not have a homogeneous population structure across worldwide locations (Brown and Tucker, 2020), therefore, it may be derived locally.

ASV2, which close to putative *Chloromonas* sp., was also commonly present in the red snow in the study area. *Chloromonas* reported to be regionally endemic distribution (Segawa et al., 2018). The broad distribution of ASV2 in the study area may disperse locally.

ASV5 dominant, which close to putative *Chlainomonas* sp. appeared only at the specific sites (S2) and was rarely observed in other red snow samples. However, we did not find any specific chemical (pH or EC) or topographical conditions at this site. The previous study showed that snow-dwelling *Chlainomonas* spp. were distributed in forested sites or close to coniferous canopies (Hoham, 1974a,b), in waterlogged snow overlying lakes (Novis et al., 2008, Procházková et al., 2018), or at other high alpine sites which were not notably wetter than the surrounding snow, nor were they located over frozen lakes (Engstrom et al., 2020). The life cycle of *Chlainomonas* includes flagellates (e.g., Procházková et al., 2018) which are important for migration processes from the ground soil to the snow surface during snow melting (Hoham and Remias, 2020). To reveal reasons for the distinct distribution of this alga in this region (when compared to other species in this dataset), measurement of physical and chemical snow parameters may be helpful.

Seasonal and spatial variations in the appearance of each algal species suggest that they have different dispersal processes, life cycles, and suitable conditions for growth on the snow surface. However, this remains largely questionable. This snowy area of the Tateyama Mountains is one of the best places in the world to study snow algae. However, global climate warming is changing the environmental conditions of this alpine area and will affect the snowpack in this unique ecosystem soon. Thus, understanding the ecology of the snow algae in this area is urgently required.

## Conclusions

This study revealed that red snow appeared extensively on the Mt. Tateyama snowfields during the melting seasons. HPLC analysis showed that the algal pigment compositions of the red snow varied spatially and seasonally and could be classified into four distinctive types (Types A–D), although they appeared visually to be almost the same color. Types A and B contained abundant astaxanthin-monoester; however, its abundance was relatively greater in Type A than in Type B. These two types occurred most commonly in the study area. Types C and D were characterized by primary carotenoids, free-astaxanthin, and astaxanthin diesters. These two types occurred only at specific sites. Phylogenic analysis using the 18S rRNA gene showed no significant difference in community structure between Types A and B, dominated by ASV1 and ASV2, which are close to *Sanguina* sp. and *Chloromonas* sp., respectively. Types C and D were dominated by ASV2, close to *Chloromonas* sp., and by ASV5, close to *Chlainomonas* sp., respectively. As both Types A and B are dominated by *Sanguina* spp., the difference in the abundance of astaxanthin between the two types was probably due to the environmental conditions and also cell maturation process as well. The pigment composition of Type C was likely induced by ASV2 because *Chloromonas* algae have abundant primary carotenoids. The pigment composition of Type D was likely derived from ASV5, *Chlainomonas* algae, which have abundant astaxanthin diesters. The factors determining the pigment composition or community structure in red snow remain uncertain. Further detailed studies examining the physical parameters such as snow water content, the amount of snow and solar radiation intensity, and chemical conditions of the snow at each site are required. The dispersal process and annual life cycle of each algal species are also important for understanding their spatial and seasonal variations.

## Data Availability Statement

Raw sequence data are available from BioProject: PRJNA726817 in the Sequence Read Archive of National Center for Biotechnology Information (NCBI; https://www.ncbi.nlm.nih.gov/sra/).

## Author Contributions

TN and NT designed the study. TN collected the samples. TN and AT conducted HPLC analyses. TN and JU performed DNA extractions and sequencing. JU, TS, and LP performed DNA data analyses. TN, NT, JU, TS, and LP wrote the manuscript. All authors contributed to the article and approved the submitted version.

## Conflict of Interest

The authors declare that the research was conducted in the absence of any commercial or financial relationships that could be construed as a potential conflict of interest.
