# Supplementary material for: Spatial and Temporal Variations in Pigment and Species Compositions of Snow Algae on Mt. Tateyama in Toyama Prefecture, Japan
Source: Front Plant Sci. 2021 Jul 5;12:689119. doi: 10.3389/fpls.2021.689119 (PMC8289405; doi:10.3389/fpls.2021.689119)
Supplement: Supplementary file 1 [file Table_1.pdf]

Supplementary Table 1. The pigment content of each sample. Pigment contents are expressed in μg/L per μg/L chlorophyll *a*.

Collected date	Sample ID	Chl	Car					
		Chl *b*	Lut	Vio	BetaC	Ast-f	Ast-e	Ast-d
Jun-15	1506_S1-2	0.265	0.044		0.009	0.088	2.690	0.021
	1506_S2-1	0.244	0.027	0.005	0.008	0.084	4.062	0.059
	1506_S2-2	0.185	0.040	0.008	0.009	0.086	3.525	0.027
	1506_S2-3	0.271	0.035	0.001	0.015	0.057	2.885	0.017
	1506_S2-3'	0.267	0.033	0.004	0.003	0.070	3.572	0.064
	1506_S3-1	0.281	0.030	0.004	0.012	0.176	5.015	0.089
	1506_S3-2	0.293	0.023	0.004	0.014	0.099	4.995	0.116
	1506_S3-3	0.370	0.044	0.005	0.008	0.213	2.867	0.061
	1506_S4-3	0.254	0.073	0.004	0.006	0.092	0.354	0.002
	1506_S5-3	0.213	0.042		0.010	0.049	0.207	0.092
	1506_S6-1	0.309	0.024	0.005	0.006	0.062	0.254	0.004
	1506_S6-2	0.317	0.022	0.004	0.007	0.064	0.226	0.004
	1506_S6-3	0.283	0.021	0.005	0.008	0.127	0.314	0.027
	1506_S6-4	0.422	0.035	0.005	0.011	0.199	5.325	0.165
	1506_S6-5	0.495	0.066	0.006	0.005	0.206	2.798	0.061
	1506_S7W-1	0.458	0.024		0.007	0.055	0.247	0.000
	1506_S7W-2	0.483	0.046		0.002		0.848	0.037
	1506_S7W-4	0.344	0.014		0.003	0.054	0.229	0.002
	1506_S7F-1	0.347	0.020	0.001	0.009	0.120	0.248	0.006
	1506_S7F-2	0.380	0.017	0.004	0.005	0.250	5.764	0.189
	1506_S7F-3	0.483	0.016	0.005	0.008	0.284	4.897	0.145
	1506_S7E-1	0.280	0.020	0.004	0.005	0.112	0.388	0.046
	1506_S7E-2	0.298	0.021	0.005	0.007	0.121	0.786	0.013
	1506_S7E-3	0.285	0.024	0.005	0.009	0.128	1.144	0.042
	1506_S7E-4	0.337	0.025	0.005	0.007	0.093	0.680	0.024
Jul-15	1507_S1-1	0.279	0.031		0.009	0.045	1.527	0.168
	1507_S4-1	0.262	0.300	0.015	0.006	0.473	0.225	
	1507_S4-3	0.245	0.232	0.011	0.007	0.443	0.350	
	1507_S5-1	0.297	0.035	0.007	0.013	0.053	0.351	0.016
	1507_S5-2	0.368	0.038	0.007	0.007	0.083	0.455	0.031
	1507_S5-3	0.232	0.043	0.009	0.017	0.076	0.544	0.019
	1507_S7W-1	0.328	0.021	0.003	0.005	0.123	0.760	0.011
	1507_S7W-2	0.313	0.023	0.004	0.008	0.124	0.693	0.005
	1507_S7W-3	0.346	0.077	0.016	0.027	0.250	2.054	0.135
	1507_S7F-1	0.334	0.019		0.008	0.082	0.581	0.006
	1507_S7F-2	0.357	0.025	0.005	0.010	0.068	0.561	0.012
	1507_S7F-3	0.338	0.023	0.004	0.012	0.058	2.829	0.085
	1507_S7E-1	0.281	0.024	0.002	0.014	0.079	2.217	0.021
	1507_S7E-2	0.299	0.018	0.003	0.008	0.073	0.696	0.032
	1507_S7E-3	0.300	0.022		0.007	0.116	0.557	0.002
	1507_S7E-4	0.327	0.022	0.000	0.010	0.067	2.651	
	1507_S7E-5	0.312	0.018		0.007	0.110	0.618	0.004
	1507_S7E-6	0.326	0.017	0.004	0.009	0.108	0.675	0.028
Jun-16	1606_S2-3	0.176	0.075	0.027	0.210	0.066	0.410	0.026
	1606_S2-4	0.186	0.066	0.005	0.444	0.060	0.681	1.502
	1606_S4-1	0.275	0.016	0.009	0.025	0.037	0.154	0.124
	1606_S4-2	0.224	0.053	0.012	0.005	0.033	0.117	0.107
	1606_S7W-2	0.301	0.029	0.000	0.000	0.033	0.123	
	1606_S7F-3	0.401	0.025	0.000	0.022	0.034	0.229	0.007
	1606_S7E-1	0.205	0.038	0.000	0.018	0.166	1.886	0.049
	1606_S7E-2	0.279	0.027	0.000	0.097	0.091	1.520	0.016
	1606_S7E-3	0.291	0.022	0.000	0.252	0.094	2.275	0.033
	1606_S7E-4	0.323	0.017	0.000	0.186	0.085	2.019	0.026
	1606_S7E-5	0.393	0.020	0.002	0.307	0.123	2.799	0.041

Abbreviations: Chl: Chlorophyll; Car: Carotenoid; Chl *b*: chlorophyll *b*; Lut: lutein; Vio: violaxanthin; BetaC: β-carotene; Ast-f: free-astaxanthin; Ast-e: astaxanthin-monoester; Ast-d: astaxanthin-diesters.
