# Supplementary material for: Spatial and Temporal Variations in Pigment and Species Compositions of Snow Algae on Mt. Tateyama in Toyama Prefecture, Japan
Source: Front Plant Sci. 2021 Jul 5;12:689119. doi: 10.3389/fpls.2021.689119 (PMC8289405; doi:10.3389/fpls.2021.689119)
Supplement: Supplementary file 2 [file Table_2.pdf]

Supplementary Table 2. The numbers of read sequence of each sample.

Collected date	sample ID	input	filtered	denoisedF	denoisedR	merged	nonchim
Jun-15	1506_S2-1	52091	40611	40375	40332	39719	31603
	1506_S2-2	67836	54456	54250	54177	53833	52833
	1506_S2-3	81638	63939	63519	63455	62655	60010
	1506_S3-2	55191	44138	44013	44075	43815	43453
	1506_S4-3	95668	76076	75860	75793	75220	73017
	1506_S5-3	77728	59258	59068	59035	58827	58677
	1506_S6-1	44169	33483	33368	33284	33077	32402
	1506_S6-2	46583	29662	29475	29398	29098	28887
	1506_S6-4	105764	81566	81303	81214	80754	79107
	1506_S6-5	80185	57341	57172	57105	56873	56285
	1506_S7W-2	73762	55965	55719	55643	55395	54663
	1506_S7W-4	89714	70195	70063	69984	69644	67773
	1506_S7F-1	109262	84081	83774	83920	83093	75175
	1506_S7F-2	90553	69870	69732	69682	69217	67401
	1506_S7F-3	74082	55912	55598	55515	54495	54273
	1506_S7E-1	64794	50086	49825	49786	49465	48370
	1506_S7E-2	86077	66285	66124	66114	65743	64764
	1506_S7E-3	83764	64994	64854	64803	64576	62739
	1506_S7E-4	62923	49542	49332	49238	48619	47431
Jul-15	1507_S1-1	59834	47758	47479	47470	45898	41458
	1507_S4-1	40238	31066	30892	30843	30535	26918
	1507_S4-3	68990	52392	52132	52082	51288	48146
	1507_S5-1	71880	52638	52446	52363	51815	50556
	1507_S5-2	49191	36807	36707	36652	36430	36263
	1507_S5-3	95598	74835	74604	74552	73953	72985
	1507_S7W-1	83697	65082	64907	64883	64487	63685
	1507_S7W-2	37545	29049	28894	28845	28706	28459
	1507_S7W-3	102904	78784	78682	78670	78467	78221
	1507_S7F-1	70181	52933	52786	52806	52302	51859
	1507_S7F-2	59637	46062	45907	45870	44753	42147
	1507_S7F-3	73342	57370	57223	57252	56466	55108
	1507_S7E-1	58455	44299	44063	43992	43083	41891
	1507_S7E-3	61843	48129	47978	47946	47162	45851
	1507_S7E-4	71747	56400	55849	55787	55012	53284
	1507_S7E-5	82126	65941	65675	65635	65372	64744
	1507_S7E-6	46776	35890	35782	35610	35015	31091
Jun-16	1606_S2-3	235868	201122	200008	199971	198728	183109
	1606_S2-4	89501	79030	78726	78810	78456	76732
	1606_S4-2	104039	89382	88642	88643	87888	84850
	1606_S7F-3	120565	103871	103585	103459	102824	101646
	1606_S7E-1	115612	96559	95272	95072	93856	91663
	1606_S7E-5	61341	53401	52918	52882	52067	49555

Total (merged) 2,568,681
