# Supplementary material for: Spatial and Temporal Variations in Pigment and Species Compositions of Snow Algae on Mt. Tateyama in Toyama Prefecture, Japan
Source: Front Plant Sci. 2021 Jul 5;12:689119. doi: 10.3389/fpls.2021.689119 (PMC8289405; doi:10.3389/fpls.2021.689119)
Supplement: Supplementary file 3 [file Table_3.pdf]

Supplementary Table 3. The relative abundance (%) of algal ASVs of each sample.

Collected date	sample ID	ASV1	ASV2	ASV3	ASV4	ASV5	ASV6	others
Jun-15	1506_S2-1	0.407	0.256	0.271	0.000	0.000	0.000	0.066
	1506_S2-2	0.783	0.031	0.123	0.000	0.000	0.000	0.063
	1506_S2-3	0.360	0.541	0.072	0.000	0.000	0.000	0.027
	1506_S3-2	0.000	0.894	0.106	0.000	0.000	0.000	0.000
	1506_S4-3	0.000	0.809	0.144	0.000	0.012	0.000	0.035
	1506_S5-3	0.809	0.191	0.000	0.000	0.000	0.000	0.000
	1506_S6-1	0.905	0.095	0.000	0.000	0.000	0.000	0.000
	1506_S6-2	0.954	0.005	0.000	0.000	0.000	0.000	0.041
	1506_S6-4	0.845	0.155	0.000	0.000	0.000	0.000	0.000
	1506_S6-5	0.160	0.470	0.145	0.000	0.173	0.000	0.053
	1506_S7W-2	0.985	0.015	0.000	0.000	0.000	0.000	0.000
	1506_S7W-4	0.987	0.000	0.001	0.012	0.000	0.000	0.000
	1506_S7F-1	0.967	0.002	0.000	0.031	0.000	0.000	0.001
	1506_S7F-2	1.000	0.000	0.000	0.000	0.000	0.000	0.000
	1506_S7F-3	0.191	0.562	0.085	0.130	0.000	0.000	0.032
	1506_S7E-1	0.993	0.001	0.000	0.005	0.000	0.000	0.001
	1506_S7E-2	1.000	0.000	0.000	0.000	0.000	0.000	0.000
	1506_S7E-3	0.830	0.028	0.013	0.104	0.000	0.000	0.025
	1506_S7E-4	0.657	0.004	0.298	0.010	0.000	0.000	0.030
Jul-15	1507_S1-1	0.757	0.057	0.023	0.000	0.000	0.000	0.163
	1507_S4-1	0.000	0.999	0.000	0.000	0.000	0.000	0.001
	1507_S4-3	0.017	0.877	0.074	0.000	0.000	0.000	0.032
	1507_S5-1	0.776	0.224	0.000	0.001	0.000	0.000	0.000
	1507_S5-2	0.908	0.092	0.000	0.000	0.000	0.000	0.000
	1507_S5-3	0.800	0.200	0.000	0.000	0.000	0.000	0.000
	1507_S7W-1	1.000	0.000	0.000	0.000	0.000	0.000	0.000
	1507_S7W-2	0.327	0.215	0.000	0.341	0.000	0.117	0.000
	1507_S7W-3	0.498	0.007	0.000	0.055	0.000	0.438	0.002
	1507_S7F-1	0.445	0.465	0.004	0.078	0.000	0.000	0.007
	1507_S7F-2	0.674	0.041	0.009	0.153	0.000	0.000	0.123
	1507_S7F-3	0.692	0.013	0.134	0.050	0.000	0.000	0.111
	1507_S7E-1	0.315	0.650	0.020	0.006	0.000	0.000	0.009
	1507_S7E-3	0.305	0.642	0.003	0.033	0.000	0.000	0.016
	1507_S7E-4	0.543	0.152	0.251	0.033	0.000	0.000	0.022
	1507_S7E-5	0.926	0.007	0.017	0.015	0.000	0.000	0.034
	1507_S7E-6	0.557	0.415	0.000	0.028	0.000	0.000	0.000
Jun-16	1606_S2-3	0.002	0.669	0.000	0.000	0.001	0.000	0.328
	1606_S2-4	0.000	0.027	0.000	0.000	0.953	0.000	0.020
	1606_S4-2	0.000	0.567	0.033	0.000	0.350	0.000	0.050
	1606_S7F-3	0.991	0.001	0.000	0.007	0.000	0.000	0.000
	1606_S7E-1	0.234	0.758	0.000	0.000	0.000	0.000	0.008
	1606_S7E-5	0.000	0.373	0.037	0.000	0.536	0.000	0.054
