# Supplementary figures and images for: Spatial and Temporal Variations in Pigment and Species Compositions of Snow Algae on Mt. Tateyama in Toyama Prefecture, Japan
Source: Front Plant Sci. 2021 Jul 5;12:689119. doi: 10.3389/fpls.2021.689119 (PMC8289405; doi:10.3389/fpls.2021.689119)

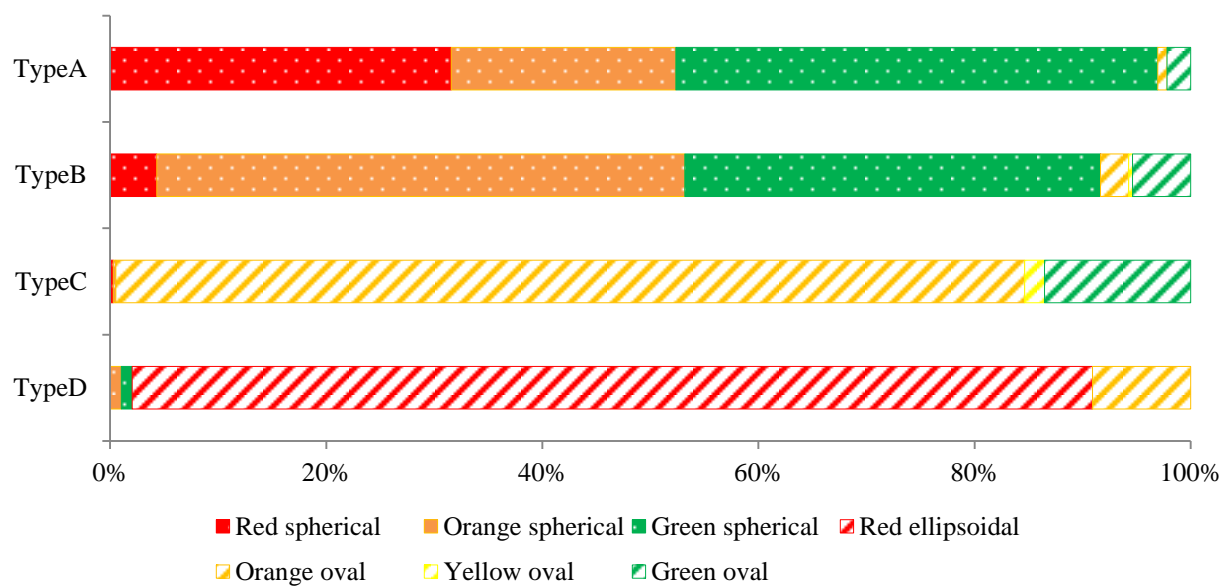

Supplementary Figure 1. The mean algal cell morphologies of each pigment types.

Supplement: Supplementary file 4 [file Image_1.pdf]
